# Diversity and functional genomic insights into antimicrobial resistance and aromatic hydrocarbon degradation genes in the Red Sea coast microbial community

**DOI:** 10.3389/fmicb.2026.1936370

**Published:** 2026-09-14

**Authors:** Riaz Ullah, Asad Karim, Sayed Sartaj Sohrab, Rania A. El-Kady, Abdalhadi Hasan, Ihsanullah Daur, Muhammad Yasir

**Affiliations:** 1 Special Infectious Agents Unit, King Fahd Medical Research Center, King Abdulaziz University, Jeddah, Saudi Arabia; 2 Jamil-Ur-Rahman Center for Genome Research, Dr. Panjwani Center for Molecular Medicine and Drug Research, International Center for Chemical and Biological Sciences (ICCBS), University of Karachi, Karachi, Pakistan; 3 Department of Medical Laboratory Sciences, Faculty of Applied Medical Sciences, King Abdulaziz University, Jeddah, Saudi Arabia; 4 Pathological Sciences Department- MBBS Program, Fakeeh College for Medical Sciences, and Fakeeh Care Group, Jeddah, Saudi Arabia; 5 Department of Medical Microbiology and Immunology, Faculty of Medicine, Mansoura University, Mansoura, Egypt; 6 Nursing Department, Fakeeh College for Medical Sciences, and Fakeeh Care Group, Jeddah, Saudi Arabia; 7 Department of Agronomy, University of Agriculture Peshawar, Peshawar, Pakistan

**Keywords:** antibiotic resistance, bacterial diversity, bioremediation, genomics, Red Sea

## Abstract

**Introduction:**

The Red Sea is a unique oligotrophic marine ecosystem characterized by high salinity, elevated temperatures, and increasing anthropogenic pressures along its coastal regions. However, coastal sediment-associated microbial communities and their functional potential, particularly regarding antimicrobial resistance and aromatic compound degradation, remain insufficiently explored.

**Methods:**

This study investigated bacterial diversity in coastal sediments from six sites along the eastern Red Sea using 16S rRNA gene amplicon sequencing and gained functional insights through genome sequencing of 21 cultured bacterial isolates.

**Results:**

Amplicon sequencing revealed diverse bacterial communities dominated by Proteobacteria, followed by Bacteroidetes and Planctomycetes. Alpha diversity indices showed no significant variation among sites, whereas beta diversity analysis demonstrated distinct community clustering influenced by environmental parameters, including temperature, salinity, and pH. Genomic analysis of 21 isolates identified multiple antimicrobial resistance genes (ARGs), conferring resistance to clinically relevant antibiotics such as beta-lactams, fluoroquinolones, and tetracyclines, alongside metal resistance determinants. Putative carbapenem resistance genes were detected in Vibrio and Idiomarina isolates. Genomic annotation predicted substantial variability in aromatic hydrocarbon degradation capacity among isolates. Genera including Marinobacter, Ruegeria, and Halomonas exhibited extensive gene interaction networks, indicating enhanced metabolic adaptability and bioremediation potential, whereas other taxa displayed limited functional connectivity, suggesting niche specialization.

**Conclusion:**

Overall, this study identifies that Red Sea coastal sediments harbor taxonomically distinct bacterial communities that carry a reservoir of ARGs and possess pollutant-degradation capabilities, providing insights for environmental health monitoring and biotechnological applications in pollution remediation.

## 1 Introduction

The Red Sea is a semi-enclosed, unique marine environment that comprises diverse ecosystems, including mangroves, coral reefs, and macroalgae (Ullah et al., 2017; Mohamed, 2018; Cochran et al., 2024). The bacterial diversity of the Red Sea is of particular scientific interest because of its oligotrophic nature, elevated temperatures, and high salinity, resulting from rapid evaporation, minimal river inflow, and low rainfall (Rasul et al., 2015). Owing to rapid urbanization along the Red Sea coast, understanding microbial communities in coastal regions and their interactions with connected ecosystems is essential for environmental health monitoring, climate regulation, and biotechnological applications (Aylagas et al., 2017).

Saudi Arabia's Red Sea coast has undergone extensive urbanization and rapid industrialization, resulting in increased industrial waste, hospital effluents, and wastewater treatment plant discharges into coastal waters (Ansari et al., 2015; Ali et al., 2017). Public and private recreational beaches further contribute as nonpoint sources of contamination (Ansari et al., 2015). Several studies have shown that such contaminants significantly disrupt marine microbial communities (Mustafa et al., 2014; Behzad et al., 2016; Ullah et al., 2017; Lal et al., 2026). Bacterial genera such as *Cyanobacteria, Prochlorococcus, Ostreococcus*, and *Gramella*, along with *Arcobacter, Pseudomonas*, and unclassified *Campylobacterales*, were previously identified in the Red Sea coastal waters (Ansari et al., 2015). Proteobacteria, Firmicutes, Fusobacteria, Bacteroidetes, and Spirochetes are also abundantly reported in the Red Sea region of Egypt, including potential human pathogens and marine oil-associated *Vibrio* species (Mustafa et al., 2014). Studies revealed the site's specific bacterial communities inhabiting the coastal, offshore, and mangrove sites of the Red Sea (Behzad et al., 2016; Ullah et al., 2017, 2019). These communities vary substantially from other oceans (Gartner et al., 2011; Da Silva et al., 2013). Previously, we identified 137 bacterial species from offshore and Red Sea coastal sites using a culture-dependent method. The study showed site-specific bacterial communities, with more antimicrobial-resistant isolates from coastal areas and multidrug-resistant strains from offshore samples (Ullah et al., 2019).

Antimicrobial resistance (AMR) poses a significant threat to human health, with approximately 5 million global deaths attributed to bacterial resistance in 2019, of which 1.3 million deaths were directly linked to AMR (Murray et al., 2022). Although AMR is a natural phenomenon, antibiotic overuse and misuse have exacerbated resistance and facilitated the spread of antibiotic resistance genes (ARGs) in marine environments, including the Red Sea (Ali et al., 2017; Ullah et al., 2019; Shao et al., 2025). In particular, the discharge of wastewater, hospital effluents, industrial waste, pharmaceuticals, heavy metals, and other anthropogenic contaminants enhances antimicrobial resistant bacteria and resistance genes into coastal ecosystems (Ali et al., 2017; Shao et al., 2025; Lal et al., 2026). Moreover, polycyclic aromatic compounds (PACs) are hazardous environmental contaminants posing severe risks to ecosystems and human health (Mallah et al., 2022). PAC contamination from industrial activities, petroleum extraction, and oil mining operations has a significant impact on public and environmental health (Abdel-Shafy and Mansour, 2016; Mallah et al., 2022). Microbial communities inhabiting the specific environment play a crucial role in degrading and reducing various aromatic pollutants (Xue et al., 2015). Genes encoding enzymes that catalyze the degradation of aromatic compounds have been identified predominantly in bacterial species such as *Achromobacter pulmonis, Achromobacter mucicolens, Pseudomonas citronellolis*, and *Comamonas thiooxydans* (Hossain et al., 2024).

Research in the Red Sea has focused mainly on coral reefs, mangroves, and fisheries, whereas sediment-associated microbial communities, particularly at coastal sites, have received comparatively less attention (Behzad et al., 2016; Ullah et al., 2019; Cochran et al., 2024). Coastal ecosystems represent important interface among marine environment, human, and animal health. Currently, expanding urbanization, recreational activities, wastewater discharge, and industrial development along the Red Sea coast may influence microbial community structure, chemical contaminants persistence, and dissemination of ARGs (Ansari et al., 2015; Ali et al., 2017; Ullah et al., 2019; Filippi and Mazzetto, 2024). From a One Health perspective, simultaneous characterization of microbial diversity, ARGs, and pollutant-degradation potential can therefore provide important information on the ecological consequences of anthropogenic pressures and their possible implications for environmental and public health (Ioannou et al., 2025).

The aim of this study was to investigate the bacterial communities in coastal sediments of the Red Sea, with a focus on the impact of apparent anthropogenic activities, and to evaluate their AMR and bioremediation potential in the context of environmental health monitoring. We utilized the integration of 16S amplicon sequencing and genome sequencing of bacterial isolates, which provided complementary strengths for comprehensively investigating bacterial communities along the Red Sea coast. The 16S amplicon sequencing is cost-effective method and efficiently profiled bacterial community composition, relative abundance, diversity, and differences among coastal sites, but lack functional resolution. Genome sequencing complements this approach and provides detailed insights into ARGs and aromatic compound degradation genes and linking them directly to specific bacterial genomes. Overall, this study provided a community-wide ecological understanding of bacterial composition on the Red Sea coast and, to our knowledge, represents the first genomic characterization of aromatic hydrocarbon degradation genes in bacterial isolates recovered from the Red Sea, revealing taxon-specific bioremediation potential.

## 2 Materials and methods

### 2.1 Sampling

Sediment samples were collected from six geographically separate sites along the eastern Red Sea coast in April and May 2016 (Figure 1). Three of these sites (CS-4, CS-6, and CS-7) were located north of Jeddah city, while the other three sites (CS-18, CS-20, and CS-22) were south of Jeddah. The site of CS-4 (27.739668, 35.446465) was clean with minimal anthropogenic activity. Site CS-6 (27.319854, 35.743117) is situated near a children's park with moderate visitor activity. Site CS-7 (26.315984, 36.397641) was a clean coastal site with an abundance of algae and coral. Site CS-18 (16.746901, 41.966400), located on the famous Red Sea Farasan Island, was apparently a clean beach. Site CS-20 (17.986386, 41.659907) represented an under-construction children's park along the coast. Algae and seaweed inhabited the site CS-22 (20.837146, 39.411522). The sampling sites were not privately owned or protected and are not located within a national park or reserve. Additionally, our sampling did not involve any endangered or protected species, and did not require any special permission for sampling.

**Figure 1:**
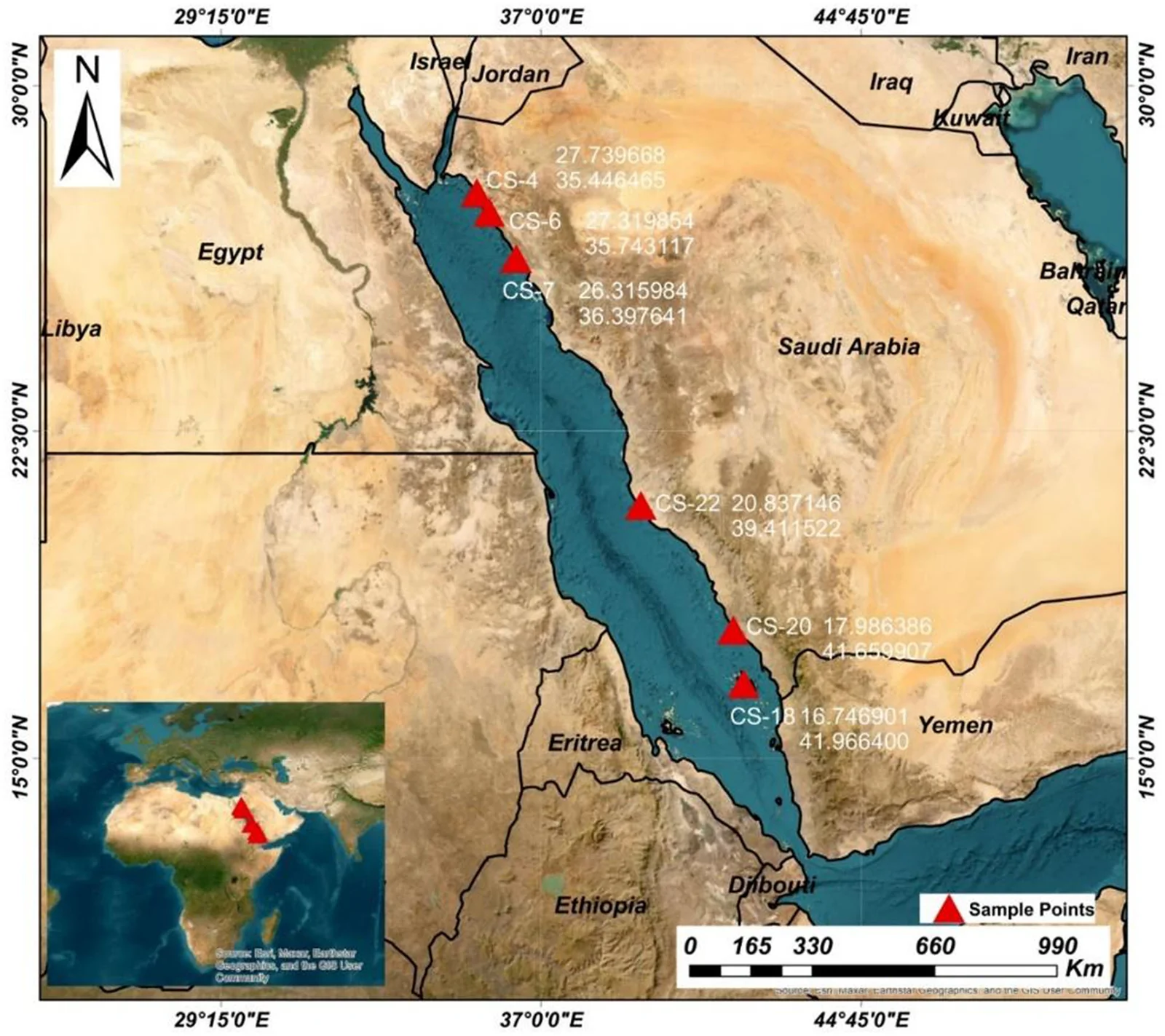
Map of the sampling sites on the Red Sea coast along with the coordinate information. Image obtained from Sentinel-2 multispectral satellite images provided by the European Space Agency (© European Space Agency).

Sediment samples were collected in triplicate at a depth of 10 cm from each site at a one-meter distance between collection points. Samples were placed in airtight, sterile zipper bags. The collected samples were transported at 4 °C and stored at −80 °C for further analysis. The environmental parameters of pH, salinity, and temperature were measured. The temperature was recorded on-site during sample collection, and the pH was measured using a portable pH meter. Salinity was measured for a 1/10 (m/v) saturated colloidal solution of sediments in water using a Martini portable meter (Martini, Australia).

### 2.2 Amplicon sequencing

#### 2.2.1 16S rRNA gene amplicon sequencing

Genomic DNA was extracted from sediment samples (300 mg) using a PowerSoil DNA Isolation Kit (Mo Bio, USA). The extracted DNA was further purified with a DNA Clean-Up Kit (MO Bio, USA). DNA quality was assessed using agarose gel electrophoresis, and DNA concentration was measured with a Qubit dsDNA HS assay kit and a Qubit Fluorometer (Invitrogen, USA). The primers of 341F and 785R were used to amplify the bacterial 16S rRNA gene (Yasir et al., 2015). Limited-cycle PCR was performed to attach dual-index barcodes and sequencing adapters to the products, using the same set of primers for sequencing. The Nextera XT protocol was adopted to normalize the libraries after sample purification with Agencourt AMPure beads (Beckman Coulter, USA). Samples libraries were loaded onto a single V3 flow cell (600 cycles) for sequencing using the MiSeq system (Illumina Inc., USA).

#### 2.2.2 16S amplicon sequencing bioinformatic and statistical analysis

The FASTQ files were uploaded to the EzBiocloud server, the paired-end reads were merged, and the sequences of the primers were removed. Sequences with a *Q*-value < 25 were filtered out, and the VSEARCH v2.30.0 program was used to de-replicate non-redundant sequences (Rognes et al., 2016). Chimeric sequences were identified using the UCHIME v4.1 program. The high-quality sequence reads were aligned to the EzBiocloud 16S database for taxonomic identification of the sequence reads. The final OTUs set was clustered at 97% similarity using the UCLUST v1.2.22q tool.

Each sample was subjected to calculating rarefaction and alpha-diversity indices, including library coverage, ACE, Chao1 value, phylogenetic diversity, and Shannon diversity index. Variability between sampling sites was evaluated using the non-parametric Mann-Whitney *U*-test. Principal Component Analysis (PCA) was performed with the default method of Singular Value Decomposition with imputation using Euclidean distance in the ClustVis tool (https://biit.cs.ut.ee/clustvis/) to explore variations in bacterial community structure across the sampling sites. In PCA analysis, the statistical method of PERMANOVA was employed to relate temperature, pH, and salinity to beta diversity using the MicrobiomeAnalyst v2.0 pipeline (Lu et al., 2023). Student's *t*-tests was used to measured taxonomic variations between different sites. UPGMA hierarchical clustering was performed using a Bray-Curtis Index to identify clustering in bacterial communities among various sampling sites. Significantly different taxa between two or more sites were identified using the Galaxy web-based tool with the Linear Discriminant Analysis (LDA) method.

### 2.3 Genome sequencing and bioinformatics analysis

Isolates were recovered from the previously described Red Sea sediment samples in our study (Ullah et al., 2019). The selection of isolates for genome sequencing was based on the relatively common marine origin species isolated at the study sites. (Ullah et al., 2019). The isolates were recovered from glycerol stock stored at −80 °C on previously described culture media and incubated at 28 °C for 48 h (Ullah et al., 2019). DNA extraction was made from the second passage of the isolates with the DNeasy UltraClean Microbial Kit (Qiagen, Germany). Isolates were identified through 16S rRNA gene sequencing, amplified using primers 27F and 1492R [19], and analyzed using the Ez-taxon database. Libraries were prepared from the genomic DNA of the isolates using a Nextera DNA Flex library preparation kit (Illumina Inc., USA), and genome sequencing was performed as described previously (Yasir et al., 2020).

The quality assessment of raw sequence reads was performed using FastQC, and sequence trimming was carried out using Trimmomatic v0.32. Contig assemblies were prepared using the SPAdes v3.15.3 program (Prjibelski et al., 2020). The assembly quality, completeness, and genomic contaminations were evaluated using CheckM v1.0.18 in KBase. The genome was annotated using BV-BRC v3.33.16 (Olson et al., 2023). The ARGs were determined using RGI 6.0.3 and ResFinder 4.4.2. A maximum-likelihood phylogenetic tree was generated using the Insert Set of Genomes into Species Tree v2.1.10 program, which was constructed from the annotated genomes of the isolates, along with closely related genomes from the NCBI microbial genome database. The phylogenetic tree was visualized using the Interactive Tree of Life tool v6.

### 2.4 Aromatic bioremediation gene screening and network visualization

Aromatic bioremediation gene analysis was conducted using R v4.3.1 [https://www.r-project.org/], employing packages including readxl, dplyr, tidyverse, igraph, ggraph, stringr, viridis, graphlayouts, and conflicted for data processing, graph construction, and visualization. Functional annotations were generated using eggNOG-mapper v2 (Cantalapiedra et al., 2021), with parameters set to -evalue 0.001, -score 60, -pident 40, -query_cover 20 and -subject_cover 20, enabling high-confidence identification of orthologs and Pfam domains. The annotation output was exported in excel format (xlsx) for downstream processing. A predefined list (Supplementary Table S1) of aromatic bioremediation genes (Neidle et al., 1991; Selvaratnam et al., 1995; Zídková et al., 2013; Setlhare et al., 2020; Eze, 2021; Mohapatra and Phale, 2021; Rojas-Vargas et al., 2023; Sun et al., 2023; Zhang et al., 2024; Yastrebova et al., 2025) was imported from a plain-text file, filtered to remove placeholder or empty entries, and stored as unique identifiers.

Gene Pfam relationships were extracted from the annotation table by selecting rows with valid preferred_name and PFAMs fields. Multiple Pfam annotations per gene were separated and aggregated to count co-occurrence frequency. Edges were defined between gene pairs sharing at least one Pfam domain or co-occurring within the same pathway. Connection types were categorized into: (i) aromatic bioremediation gene pairs, (ii) aromatic–other gene pairs, and (iii) other gene pairs. Edge weights were assigned hierarchically (0.02, 0.015, 0.01) to prioritize interactions involving aromatic genes.

Graphs were constructed using igraph, and network layouts were calculated using stress majorization via the graphlayouts package. For each node (gene), degree centrality and betweenness were computed. Genes were classified as “Hub” if their degree exceeded the network median, or as “Peripheral” otherwise. Node color was used to indicate function: red for aromatic genes, blue for hub genes, and green for peripheral genes. Edge colors represented interaction type: purple for aromatic-aromatic pairs, orange for aromatic-other, and gray for other-other connections. Gene labels, particularly for aromatic genes, were adjusted using ggrepel to avoid overlapping and enhance clarity.

## 3 Results

Environmental parameters, including temperature, pH, and salinity, were measured for the coastal samples (Supplementary Table S2). The highest temperature was observed at the Red Sea south coastal sites (CS-18, CS-20, and CS-22), ranging from 33.0 °C to 33.5 °C, while the lowest temperature was recorded at CS-4, located on the Red Sea north coast (Supplementary Table S2). The highest salinity was recorded at CS-7, while the lowest salinity (3.1 ± 0.1%) was observed at CS-4. The pH ranged from 7.6 to 8.0 for all samples, except for CS-20, which had a higher pH (9.9 ± 0.03) than the other study sites (Supplementary Table S2).

### 3.1 Bacterial diversity analysis

#### 3.1.1 Alpha and beta diversity analysis

The replicated samples from each site generated over 30,000 high-quality reads with excellent library coverage of more than 98.8%. The rarefaction curves constructed from OTUs tended to reach saturation, demonstrating that sequencing reads reliably covered the bacterial diversity in the coastal sites (Supplementary Figures S1). Alpha diversity indices, including species richness metrics (Chao1, observed species, and ACE), and the Shannon diversity index, showed variation between sampling sites. However, these variations were statistically non-significant (*p* > 0.05; Supplementary Figures S2A–D). The highest Chao1, ACE, and Shannon index values were recorded at CS-22 (south of Jeddah), followed by CS-4 and CS-6 at the northern coast sites. The lowest bacterial diversity was observed at the Farasan Island site (CS-18); (Supplementary Figure S2D).

PCA at the OTU level revealed distinct bacterial community assemblages across samples. PC1 (28.0%) and PC2 (23.9%) explained the variance in bacterial communities. Significant taxonomic diversity (*p* = 0.01) was observed among samples, and close clustering was not found (Supplementary Figure S3A). Sites CS-18 and CS-20 were relatively clustered compared to CS-7, CS-22, and CS-4 (Supplementary Figure S3A). Bray-Curtis dendrogram analysis revealed significant phylogenetic differences among sampling sites (Supplementary Figure S3B). Sub-clustering of sites CS-4 and CS-6 was noted adjacent to the CS-22 cluster, while CS-18 and CS-20 formed a separate cluster (Supplementary Figure S3B). Furthermore, significant differences (*p* < 0.05) were observed in beta diversity in relation to temperature, pH, and salinity (Supplementary Figure S4A–C). The bacterial communities from the three sites with temperatures ranging from 33.0 °C to 33.5 °C clustered separately from those at sites with temperatures below 28.4 °C. The high-salinity site, CS-7, also clustered separately from the others in the PCA analysis, as determined by the PERMANOVA statistical method.

#### 3.1.2 Taxonomic analysis of bacterial communities

The composition and relative abundance of the bacterial phyla varied among sampling sites. Proteobacteria was the most dominant phylum, followed by Bacteroidetes, Planctomycetes, Actinobacteria, Acidobacteria, Cyanobacteria, Firmicutes, Verrucomicrobia, and Gemmatimonadetes along the Red Sea coast (Figure 2A). Proteobacteria were significantly (*p* < 0.05) abundant at site CS-18 (87.8 ± 0.1%), followed by CS-7 (62.7 ± 0.3%) and CS-20 (60.8 ± 0.3%). A relatively high abundance (*p* < 0.05) of Bacteroidetes was noted in samples from site CS-6 (19.8 ± 0.3%), with ≥10% in other samples, except for Farasan beach (CS-18); (1.8 ± 0.1%). Planctomycetes were predominantly detected in samples from CS-4 (10 ± 0.5%) and CS-22 (11 ± 0.2%) sites (Figure 2A), and the variation in percentage relative abundance among the sampling sites was significant (*p* = 0.005).

**Figure 2:**
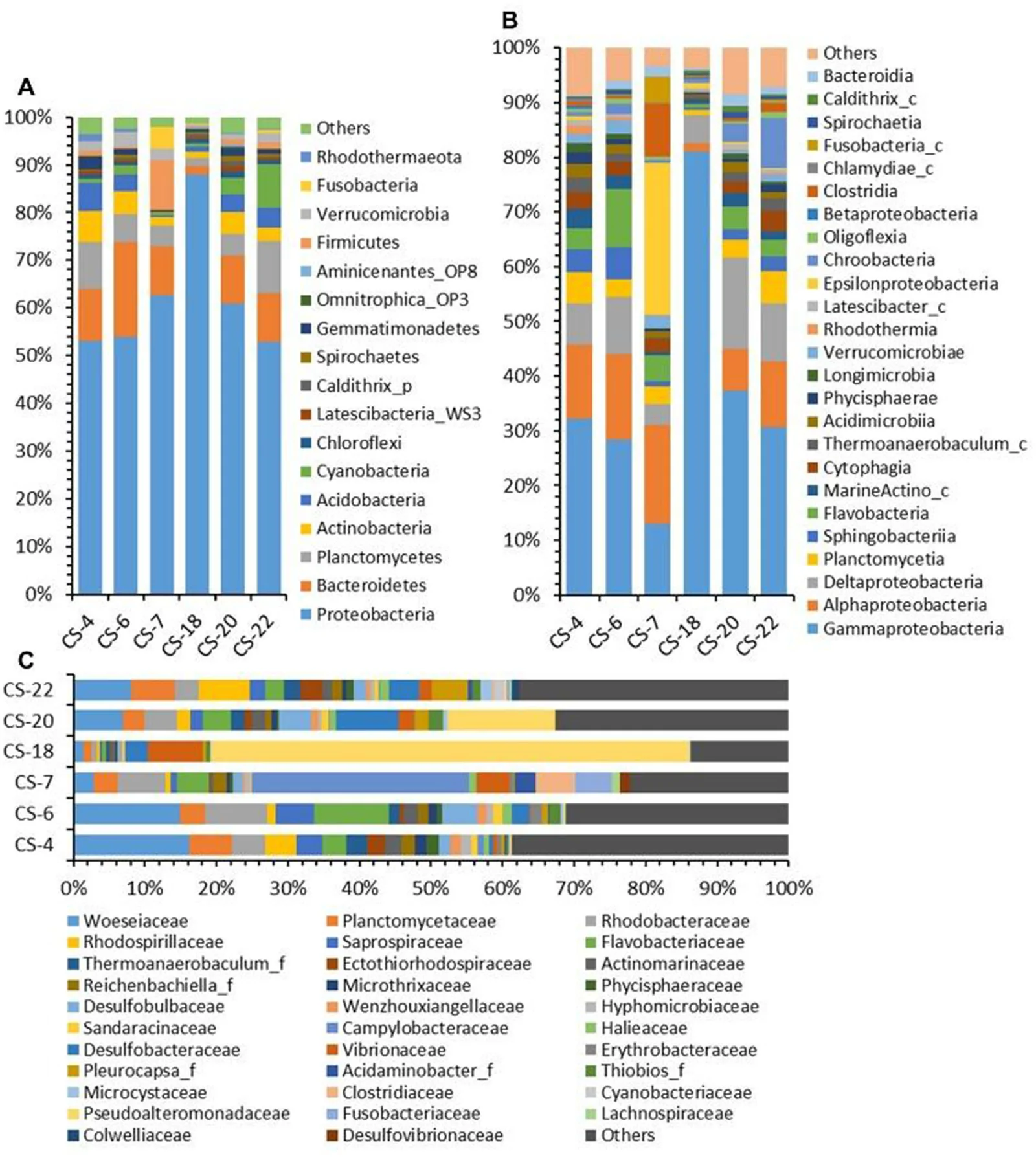
Percentage relative abundance of bacterial communities at the Red Sea coastal sites at the taxonomic levels of **(A)** phylum, **(B)** class, and **(C)** family.

The phylum Proteobacteria was primarily composed of Gammaproteobacteria, followed by Alphaproteobacteria, Deltaproteobacteria, and Epsilonproteobacteria (Figure 2B). The Bacteroidetes were mainly comprised of Flavobacteria, Sphingobacteria, and Cytophagia. Planctomycetia mainly dominated Planctomycetes. Other taxonomic classes, such as Verrucomicrobiae, Thermoanaerobaculum, Acidimicrobiia, Longimicrobia, and Phycisphaerae, were found at relative abundances of >1% (Figure 2B). The distribution of classes varied among different sampling sites. For example, Gammaproteobacteria was prevalent in all the studied sites, with the highest abundance in the CS-18 sampling site (80.3 ± 0.2%), while the abundance of Epsilonproteobacteria and Clostridia was relatively high at CS-7 (Figure 2B).

The family Woeseiaceae, from the class Gammaproteobacteria, was identified as a core family in all sampling sites, with a detection range of 1.3 to 14.5% (Figure 2C). Rhodospirillaceae, Planctomycetaceae, Flavobacteriaceae, and Rhodobacteraceae were also dominant in the coastal sites (Figure 2C). The family Woeseiaceae was significantly (*p* < 0.05) abundant in CS-4 and CS-6 at the north coast of the Red Sea compared to other sampling sites. The family Pseudoalteromonadaceae was highly abundant at the CS-18 site (66.3 ± 1%), followed by the CS-20 site (14.4 ± 0.8%). Campylobacteraceae was detected at a relatively high abundance in the CS-7 site (27.4 ± 0.9%) compared to other sites, detected at less than 1% relative abundance (Figure 2C).

#### 3.1.3 Differentially abundant taxa among the coastal sites

LDA effect size (LEfSe) identified differentially abundant taxa in the six sampling sites. Several bacterial taxa were distinguished to at least one sampling site at a default logarithmic LDA value of 2.0. The cladogram represented taxa with an LDA value greater than 2 (Figure 3A, B). The phylum Acidobacteria was significantly more abundant in CS-4. Planctomycetes and Cyanobacteria were found at significant abundance in CS-22 (Figure 3B). Alphaproteobacteria (LDA value > 5) was substantially more abundant in CS-7. Gammaproteobacteria (LDA value > 5) and Deltaproteobacteria were more abundant in CS-18 and CS-20, respectively (Figure 3B). Orders such as Chromatiales and Alteromonadales (LDA value > 5) were significantly enriched in CS-6 and CS-18, respectively. Families, such as Pseudoalteromonadaceae (LDA value > 5), were found considerably more abundant in CS-18 (Figure 3B), while the genus *Pseudoalteromonas* (LDA value > 5) was significantly more abundant in CS-18 (Figure 3B).

**Figure 3:**
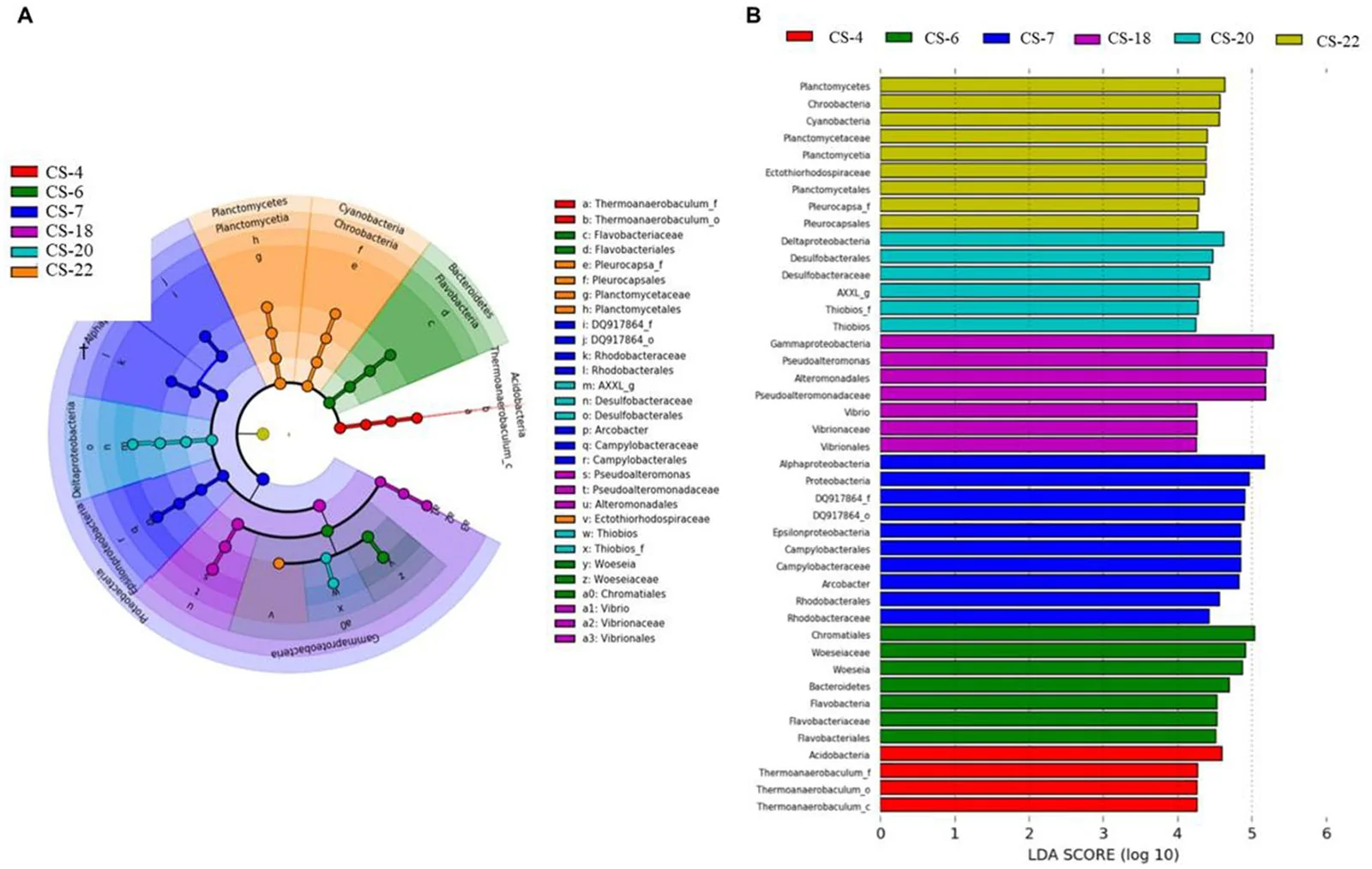
Linear discriminant analysis effect size (LEfSe) analysis to identify differentially abundant taxa across the Red Sea coast. **(A)** A cladogram illustrates the evolutionary relationship between significant taxa, and † represent Alphaproteobacteria. **(B)** Biomarker taxa with linear discriminant analysis (LDA) score across the sites. Bar colors indicate the sampling sites, while bar length represents the discriminatory effect size.

### 3.2 Bacterial isolates genomic analysis

#### 3.2.1 Isolates taxonomy and ARGs distribution in the genomes

The 21 sequenced isolates were classified into 11 genera from 16S rRNA gene sequences and genome analysis. Five of the isolates were classified as *Idiomarina*, three as *Halomonas*, and two as each of the *Marinobacter, Oceanisphaera*, and *Cobetia* species (Figure 4). Species-level identification was not found for most isolates recovered from the Red Sea. The average G+C content of the isolates ranged from 40.8 to 64.0%, and the genomic features of the isolates are summarized in Supplementary Table S3. In the phylogenetic analysis based on genomic annotation, *Epibacterium scottomollicae* CS14-81, *Ruegeria* sp. CS12-79, and *Roseovarius* sp. OS2T-32 was clustered (Figure 4). The isolates from *Cobetia* were phylogenetically linked in a distinct clade with isolates from *Halomonas*. The *Idiomarina* isolates were clustered with *Idiomarina zobellii, Idiomarina piscisalsi*, and *Idiomarina loihiensis* (Figure 4).

**Figure 4:**
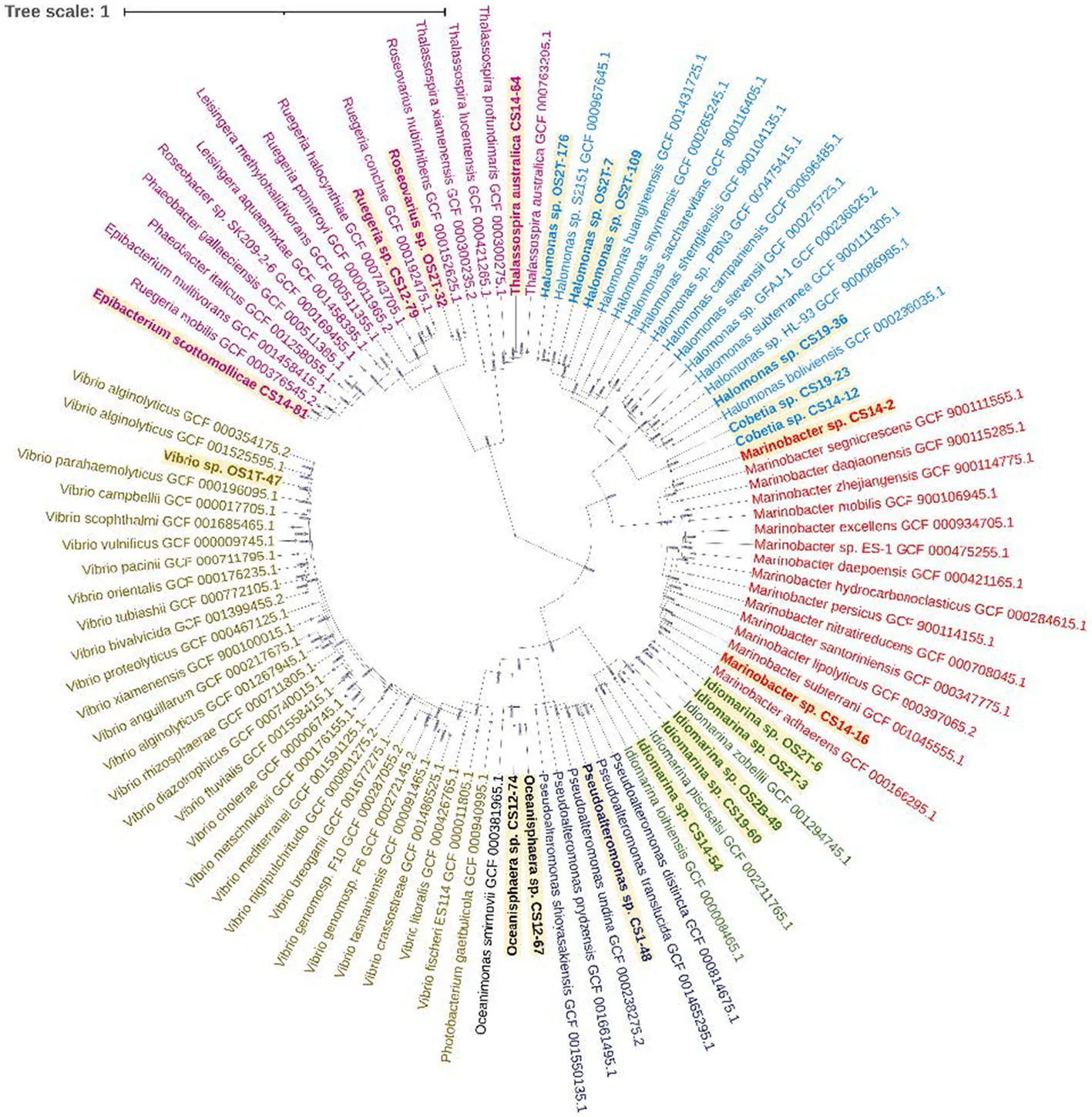
Maximum likelihood phylogenetic analysis based on genome annotation of the isolates from the Red Sea with closely related genomes of bacterial species retrieved from GenBank. Bold font highlights the genomes from this study.

In the genomes of Red Sea isolates, 16 ARGs and their variants were identified, primarily conferring resistance to beta-lactams, fluoroquinolones, tetracyclines, diaminopyrimidines, and peptide antibiotics (Figure 5). The predominant resistance mechanisms in these isolates involved antibiotic efflux, followed by antibiotic inactivation, target alteration, and target replacement. Additionally, two genes associated with resistance to disinfectants and antiseptics, *qacG* and *qacJ*, were detected, with *qacG* commonly found in isolates (Figure 5).

**Figure 5:**
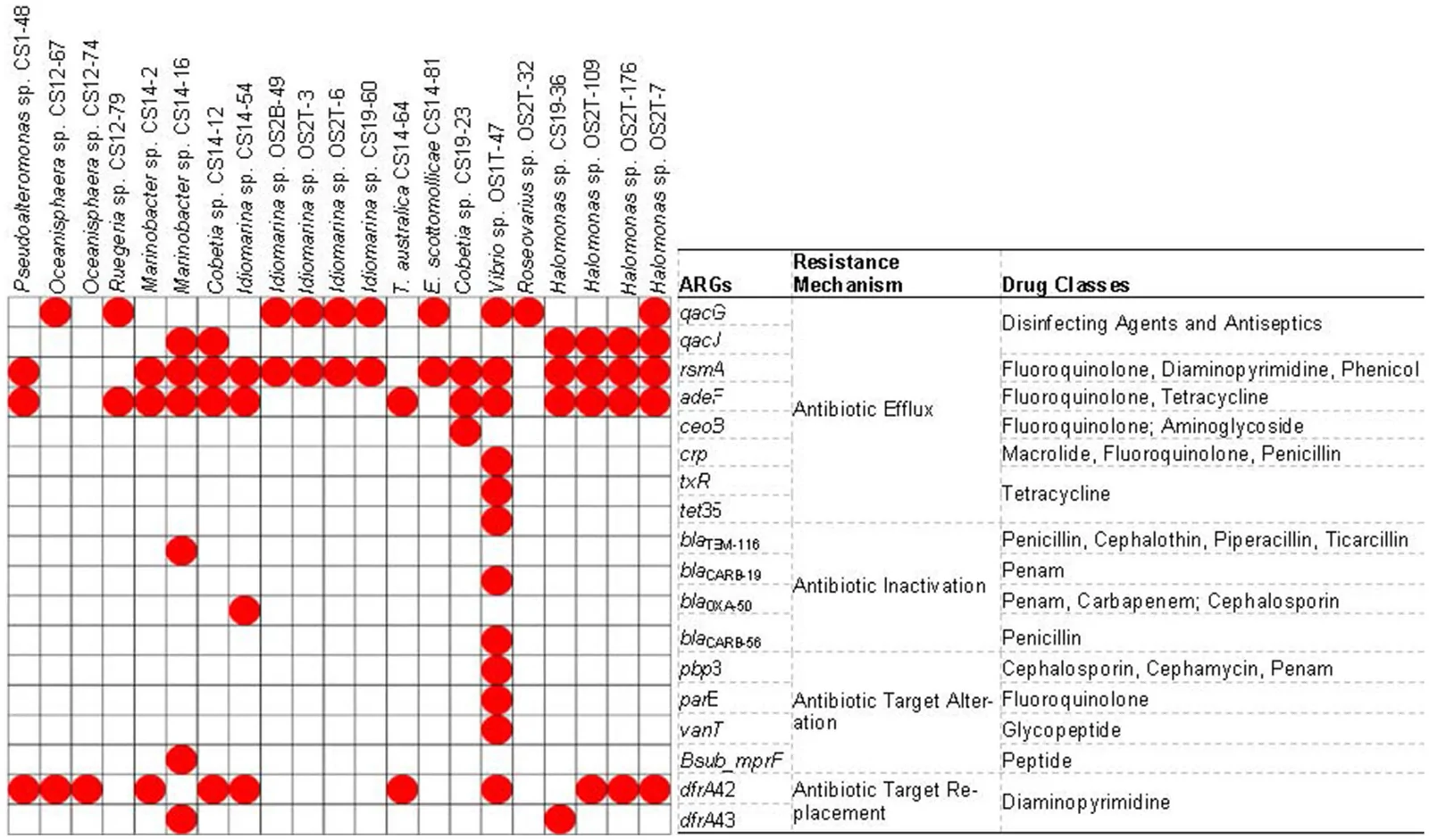
Distribution of antimicrobial resistance genes (ARGs) identified in the genome sequences of Red Sea isolates. The white box indicates that the respective gene was not detected.

An average of 3.3 ± 2.4 ARGs and variants were identified per isolate. Seven isolates carried ≥4 ARGs. The highest number of 12 ARGs was identified in the *Vibrio* sp. OS1T-47 followed by six ARGs in the *Marinobacter* sp. CS14-16 (Figure 5). The resistance-nodulation-cell division (RND) antibiotic efflux pump genes *rsmA* and *adeF* were detected in more than 70% of the isolate genomes, conferring resistance to fluoroquinolone, diaminopyrimidine, phenicol, and tetracycline antibiotics. Genes such as *bla*_IMP − 44_, *bla*_CARB − 19_, and *bla*_OXA − 50_, which produce carbapenem antibiotic resistance, were identified in isolates of *Vibrio* sp. OS1T-47, and *Idiomarina* sp. CS14-54. The trimethoprim-resistant dihydrofolate reductase *dfrA*42 gene was found in more than 50% of isolates, but the identity percentage was lower. The glycopeptide resistance gene cluster, *vanT*, was found in the isolate *Vibrio* sp. OS1T-47 (Figure 5).

#### 3.2.2 Metal resistance in the isolates from genome annotation

Genomic annotation revealed the presence of diverse metal resistance genes encoding 29 distinct proteins in Red Sea isolates, with each isolate carrying 11 and 19 metal resistance-associated proteins (Supplementary Figure S5). Commonly detected proteins included copper homeostasis protein (CutE), magnesium and cobalt efflux protein (CorC), copper-translocating P-type ATPase (CIA), a multidrug resistance transporter from the Bcr/CflA family (ClfA), mercuric ion reductase (MIR), and multicopper oxidase (MO).

Proteins associated with copper homeostasis were the most prevalent among these isolates (Supplementary Figure S5). The cobalt-zinc-cadmium resistance protein (CzcD) was present in more than 50% of the isolates and all species from the *Halomonas* and *Marinobacter* genera. Mercuric ion reductase was commonly detected in *Idiomarina* and *Marinobacter* species. Chromate transport protein (ChrA), linked to resistance against chromium, was identified in 14 genomes and was commonly found in *Halomonas* species (Supplementary Figure S5). Notably, proteins involved in suppressing copper sensitivity (ScsB, ScsC, and ScsD) and the periplasmic divalent cation tolerance protein (CutA) were mainly detected in *Idiomarina* species (Supplementary Figure S5).

#### 3.2.3 Aromatic bioremediation genes analysis

The metabolic potential and genetic network structures of 21 bacterial isolates were analyzed to assess their bioremediation capabilities, with a particular focus on aromatic hydrocarbon degradation (Supplementary Table S4). *Pseudoalteromonas* sp. CS1-48 (Figure 6A) exhibited limited aromatic metabolism, possessing only two aromatic bioremediation genes. One of these genes (*pheA*) formed interaction networks with other genes, while the second gene (*xylE*) remained unconnected. The overall genetic network of CS1-48 comprised 516 nodes, including 256 hub genes and 260 peripheral genes, suggesting a decentralized structure with weak pathway integration between bioremediation genes and other genetic elements. In contrast, *Oceanisphaera* sp. CS12-67 (Figure 6B) displayed stronger bioremediation potential, containing eight aromatic genes (*benA, catA, benC, catB, catC, benB, benD*, and *pheA*) that formed 3,521 interactions with peripheral genes within a 460-node network. Similarly, *Oceanisphaera* sp. CS12-74 (Figure 6C) carried a single *pheA* gene, which established 209 direct interactions within a broader 21,135 interaction network spanning 210 nodes. The interactions were distributed among 100 hub genes and 109 peripheral genes. Among the isolates, *Ruegeria* sp. CS12-79 (Figure 6D) exhibited the most robust metabolic adaptability, with eight aromatic genes (*xylA, xylB, xylF, xylH, catB, pheA, nahG*, and *nagK*) forming 4,114 interactions within the largest observed genetic network (809 nodes).

**Figure 6:**
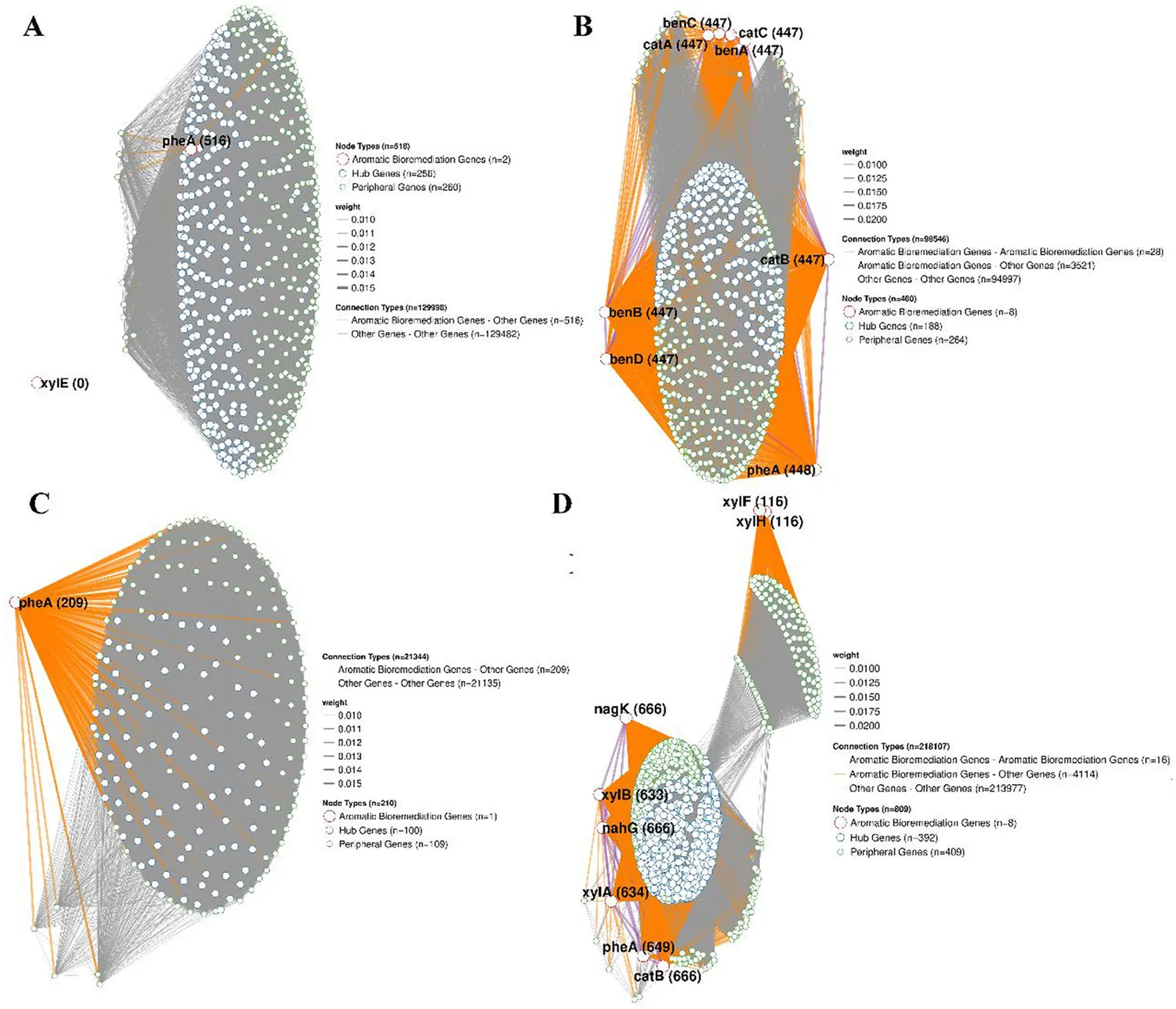
Genetic networks of **(A)** *Pseudoalteromonas* sp. CS1-48, **(B)** *Oceanisphaera* sp. CS12-67, **(C)** *Oceanisphaera* sp. CS12-74, and **(D)** *Ruegeria* sp. CS12-79. *Ruegeria* sp. CS12-79 has the largest network (809 nodes and 4,114 interactions), while *Pseudoalteromonas* sp. CS1-48 shows weak integration (516 nodes).

*Marinobacter* sp. CS14-2 (Supplementary Figure S6A) contained a single bioremediation gene (*pheA*) that established 506 interactions with other genes. In comparison, *Cobetia* sp. CS14-12 (Supplementary Figure S6B) harbored four aromatic genes (*pheA, xylB, xylG*, and *xylE*). While *xylE* showed no interactions, the remaining genes collectively formed 1,045 interactions with other genes. *Marinobacter* sp. CS14-16 (Supplementary Figure S6C) demonstrated the highest aromatic bioremediation gene count (10 genes: *dmpP, dmpK, xylE, xylG, pheA, xylH, dmpN, dmpO, dmpM*, and *xylJ*). These genes interacted internally 45 times and formed 5,926 external interactions, indicating extensive involvement in genetic networks. In contrast, *Idiomarina* sp. CS14-54 (Supplementary Figure S6D) contained only one aromatic gene (*pheA*2) with 427 interactions.

Among the isolates analyzed in Supplementary Figure S7, *T*. *australica* CS14-64 (Supplementary Figure S7A) contained two aromatic bioremediation genes, *xylB* and *pheA*, that formed a single direct interaction with each other, while 891 interactions were established with other genes. The genetic network included 450 nodes, with 202 hub genes and 246 peripheral genes. *E. scottomollisae* CS14-81 (Supplementary Figure S7B) exhibited a highly complex genetic network, containing nine aromatic genes (*xylG, xylF, xylH, pheA, nahG, xylA*, and *xylB*) that formed 2,838 interactions with other genes. This isolate had 788 total nodes, including 365 hub genes and 416 peripheral genes. *Cobetia* sp. CS19-23 (Supplementary Figure S7C) harbored three aromatic genes (*xylG, xylB, pheA*), forming 598 interactions within a network of 326 total nodes, 159 hub genes, and 164 peripheral genes. Meanwhile, *Halomonas* sp. CS19-36 (Supplementary Figure S7D) exhibited a relatively extensive genetic network, with five aromatic genes (*pheA, pheA2, xylB, benD, xylC*) forming 10 internal interactions and 2,567 external interactions within a 539-node network, consisting of 262 hub genes and 272 peripheral genes.

*Vibrio* sp. OS1T-47 exhibited minimal interaction among aromatic genes, with only one direct interaction but 1,097 interactions with other genes (Supplementary Figure S8A). It contained 557 nodes, two aromatic genes (*pheA* and *nagK*), 270 hub genes, and 285 peripheral genes. *Idiomarina* sp. OS2B-49 (Supplementary Figure S8B) showed the lowest interaction complexity, with no direct interactions among aromatic genes and only 402 interactions with other genes. Its network was the smallest, with 403 nodes, a single aromatic gene (*pheA2*), 188 hub genes, and 214 peripheral genes. On the other hand, *Halomonas* sp. OS2T-109 (Supplementary Figure S8C) and *Halomonas* sp. OS2T-176 (Supplementary Figure S8D) exhibited greater genetic complexity, each possessing three interactions among aromatic genes and a broader genetic network. *Halomonas* sp. OS2T-109 established 1,474 interactions, spanning 513 total nodes, while *Halomonas* sp. OS2T-176 displayed a similar structure with 1,475 interactions and 514 total nodes.

*Roseovarius* sp. OS2T-32 (Supplementary Figure S9A) exhibited high genetic connectivity, with three aromatic genes (*pheA, nagK, nahG*) forming 1,735 interactions within a 600-node network, including 268 hub genes and 329 peripheral genes. In contrast, *Idiomarina* sp. OS2T-3 (Supplementary Figure S9B) and *Idiomarina* sp. OS2T-6 (Supplementary Figure S9C) displayed minimal connectivity, with no direct interactions between aromatic genes. *Idiomarina* sp. OS2T-3 had 424 interactions, while *Idiomarina* sp. OS2T-6 showed 425 interactions, each forming networks of 425 and 426 nodes, respectively, with only a single aromatic gene, *pheA*2. Lastly, *Halomonas* sp. OS2T-7 (Supplementary Figure S9D) demonstrated genetic complexity comparable to *Roseovarius* sp. OS2T-32, with three aromatic genes (*xylB, benD, pheA*) forming 1,477 interactions within a 514-node network, including 250 hub genes and 261 peripheral genes. Among the 21 bacterial genomes, aromatic-degrading genes were absent in *Idiomarina* sp. CS19-60.

## 4 Discussion

Coastal sediments integrate the effects of multiple environmental pressures by accumulating organic pollutants, metals, and microorganisms over time (Ali et al., 2017; Al-Mutairi and Yap, 2021). Exploration of marine bacterial communities is essential because they play a vital role in the food web, environmental health, and biogeochemical cycles of aquatic ecosystems. The Red Sea coastline undergoes increasing urban, industrial, tourism, and recreational development, monitoring sediment-associated microbial communities, antimicrobial resistance determinants, and pollutant-degradation genes may provide complementary indicators of environmental health (Ansari et al., 2015; Ullah et al., 2017; Filippi and Mazzetto, 2024). This study reports on the bacterial diversity of sediments from the eastern coast of the Red Sea using 16S amplicon sequencing and performed genomic analysis of the isolates. Alpha-diversity analysis revealed no significant differences among the sites; however, a difference in beta diversity was observed in the clustering of bacterial communities across the study sites. The observed differences in beta diversity among sampling sites are consistent with previous reports of spatial heterogeneity within Red Sea sediment, mangrove, and planktonic microbial communities (Mustafa et al., 2014; Pearman et al., 2017; Ullah et al., 2017).

Proteobacteria was the most dominant bacterial phylum observed on the Red Sea coast. Similar to this study, a high relative abundance of Proteobacteria has been reported from the Egyptian coast of the Red Sea, the Yellow River Delta, and coastal wetlands in China (Mustafa et al., 2014; Li et al., 2019). The relatively high abundance of Proteobacteria in this study may be attributed to the rapid growth rate and robust metabolic capabilities of its members (Behera et al., 2017). Other frequently detected phyla included Planctomycetes, Bacteroidetes, Gemmatimonadetes, Acidobacteria, Cyanobacteria, and Actinobacteria. These phyla have also been previously reported in China's coastal wetlands, the Yellow River Delta, and other marine sites (Ansari et al., 2015; Zhang et al., 2019). Proteobacteria, together with Bacteroidetes and other major bacterial groups, have been reported in the Red Sea coastal waters and sediments (Mustafa et al., 2014; Pearman et al., 2017; Hempel and Fruhe, 2025). Previous studies from the Red Sea have also demonstrated substantial site-specific variation among coastal, offshore, and mangrove-associated microbial communities (Ansari et al., 2015; Pearman et al., 2017; Ullah et al., 2019). As observed in previous studies, Proteobacteria demonstrated a positive correlation with environmental factors such as pH and salinity but a negative correlation with temperature (Hinthong et al., 2024). However, this study did not correlate ecological factors in detail because no detailed analysis of the environmental factors was performed, which is a limitation of this study.

Planctomycetes, along with Proteobacteria, were the dominant phyla across all sampling sites. Proteobacteria actively participate in the marine nitrogen cycle and are known to degrade sedimentary organic nitrogen efficiently (Gutierrez, 2019; Hinthong et al., 2024). Planctomycetes contribute to biogeochemical processes such as methane oxidation, anaerobic ammonium oxidation, and carbon recycling in collaboration with other bacteria and archaea (Tang et al., 2009). Among Proteobacteria, the classes Gammaproteobacteria and Alphaproteobacteria were dominant. Gammaproteobacteria included obligate hydrocarbonoclastic bacteria, represented by genera such as *Oleiphilus, Alcanivorax, Oleibacter, Cycloclasticus, Thalassolituus, Neptunomonas*, and *Oleispira*, which play a crucial role in oil bioremediation in marine environments (Gutierrez, 2019). Similarly, members of the *Roseobacter* lineage within Alphaproteobacteria have been shown to degrade hydrocarbons in marine environments (Buchan et al., 2019).

Cyanobacteria and Chloroflexi, known for their carbon- and nitrogen-fixing capabilities, were also detected (Klawonn et al., 2016). Chloroflexi, which play a role in breaking down complex organic compounds, sulfur cycling, and bioremediation, were found only at site CS-20, possibly indicating contamination with organic compounds and sulfur (Speirs et al., 2019; Zheng et al., 2021; Freches and Fradinho, 2024). Bacteroidetes, essential for organic aggregate association in freshwater phytoplankton blooms (Riemann and Winding, 2001; Tang et al., 2009), were represented by *Flavobacteria* and *Cytophaga*, which were present at all sites except CS-18, consistent with findings from Wadden Sea sediments (Llobet-Brossa et al., 1998).

Certain microbial groups exhibited site-specific patterns. For example, Pseudoalteromonadaceae was significantly dominant at sites CS-18 and CS-20. These Na+-requiring chemoorganotrophs, known for their ability to grow in high sodium chloride concentrations, were prominent (Ivanova et al., 2004). Family Woeseiaceae, found predominantly in all sampling sites, are considered core microbial community members in diverse marine sediments and contribute to N_2_O emissions (Mußmann et al., 2017). Campylobacteraceae, detected at relatively high abundance in site CS-7, inhabit food animals, ground, and surface water. These bacteria also colonize the intestinal mucosal surfaces, oral and urogenital cavities, of humans, birds, and animals (Lastovica et al., 2014). Furthermore, Campylobacteraceae are involved in nitrogen cycling, previously reported in some estuaries (Huang et al., 2021). Gammaproteobacteria, Pseudoalteromonas, Alteromonadales, and Pseudoalteromonadaceae, which degrade marine organic nitrogen, were notably abundant at site CS-18 (Thurber et al., 2009). Thermoanaerobaculales, Thermoanaerobaculum, and Thermoanaerobaculaceae were distinguished at site CS-4. These chemoheterotrophic bacteria grow on organic acids, sugar, and protein compounds (Dedysh et al., 2015). Thermoanaerobaculum members are recognized for their capacity to degrade organic acids and biomass, even at elevated temperatures (Losey et al., 2013). Their presence at site CS-4 may be due to the discharge of dense organic acids.

Antimicrobial resistance in marine environments is a concern and has been increasingly documented in the literature, particularly from the perspective of One Health (Dewi et al., 2021). This study identified ARGs associated with producing resistance to carbapenems, cephalosporins, quinolones, and peptide antibiotics in the genomes of the Red Sea isolates. The detection of carbapenem resistance in marine bacteria is an emerging threat to public health and has been documented in recent studies from marine ecosystems (Dewi et al., 2020, 2021). Coastal environments receive contaminants from various human activities such as wastewater discharge, recreational use, other anthropogenic sources and can act as reservoirs for ARGs (Ali et al., 2017; Lal et al., 2026). Detecting carbapenem-resistance genes in the Red Sea coastal sediments supports the importance of monitoring these environments for AMR (Dewi et al., 2020, 2021). However, the findings do not indicate an immediate clinical threat because the isolates recovered from the Red Sea coast were environmental bacteria, and no clinically important pathogenic bacteria was isolated. Moreover, several resistance determinants, including certain OXA-type beta-lactamases, may be intrinsic or species-associated rather than recently acquired resistance genes (Takebayashi et al., 2021). Moreover, genomic presence does not alone establish phenotypic carbapenem resistance or demonstrate an immediate clinical risk (Rose et al., 2023; Hu et al., 2024). However, environmental bacteria carrying intrinsic or acquired ARGs may contribute to the broader environmental resistome (Larsson and Flach, 2022; Raziq et al., 2026).

Consistent with previous studies, most isolates in this study carried antibiotic efflux pumps such as RND that produce multidrug resistance (Li et al., 2015). Contamination from anthropogenic activities, sewage, and industrial waste on the Red Sea Saudi coast may be a factor in the distribution of these ARGs in the bacterial isolates identified via genome sequencing (Ali et al., 2017). The findings of this study revealed the co-occurrence of ARGs and metal resistance genes. These findings were consistent with previous results in which a positive correlation had been reported between ARGs and metal resistance genes (Neethu et al., 2015). The co-occurrence of ARGs and metal-resistance determinants may also be environmentally relevant. Heavy metals and antimicrobial agents can exert distinct but overlapping selective pressures on environmental bacterial populations (Wang et al., 2021; Gillieatt and Coleman, 2024). Metal exposure may indirectly promote the persistence of AMR when the corresponding resistance determinants are genetically linked on the same or associated mobile genetic elements, known as co-resistance (Pal et al., 2015; Gillieatt and Coleman, 2024). Because metals are persistent environmental contaminants, metal contamination may maintain antimicrobial resistance even where antibiotic concentrations are relatively low or antibiotic selective pressure is absent (Baker-Austin et al., 2006; Murray et al., 2024).

The results of this study highlight significant variability in the genetic network structures and metabolic capabilities of bacterial isolates involved in the degradation of aromatic hydrocarbons. The diversity observed in both the number of aromatic bioremediation genes and their interaction networks suggests varying degrees of metabolic efficiency, adaptation to environmental contaminants, and functional specialization among the isolates. A clear trend emerged where isolates with more extensive genetic interaction networks demonstrated higher potential for aromatic compound degradation. For instance, *Ruegeria* sp. CS12-79, *E. scottomollisae* sp. CS14-81, *Marinobacter* sp. CS14-16, and *Halomonas* sp. CS19-36 exhibited the largest and most complex interaction networks, containing multiple aromatic bioremediation genes (≥8 genes) with thousands of interactions with other genetic elements. This high degree of connectivity suggests that these isolates may have enhanced metabolic plasticity, allowing them to degrade a broader range of aromatic hydrocarbons.

In contrast, isolates such as *Idiomarina* sp. CS14-54, *Idiomarina* sp. OS2T-3, and *Idiomarina* sp. OS2T-6 had a single aromatic bioremediation gene and limited interactions, indicating a more restricted metabolic potential. The number of hub genes also appeared to be a critical factor influencing the efficiency of bioremediation networks. In a gene network, hub genes are those that interact extensively with multiple other genes and typically play a crucial role in biological processes and gene regulation (Yu et al., 2017). On the other hand, peripheral genes influence biological processes indirectly by interacting with core genes and regulating them through gene interaction networks (Fóthi et al., 2022).

Isolates such as *E. scottomollisae* sp. CS14-81 (365 hub genes) and *Halomonas* sp. CS19-36 (262 hub genes) exhibited a higher number of hub genes, which likely contributed to their well-integrated metabolic systems. On the other hand, isolates with fewer hub genes, such as *Idiomarina* sp. OS2B-49 and *Idiomarina* sp. OS2T-3, had minimal genetic interactions, suggesting a lower efficiency in aromatic hydrocarbon degradation. Additionally, some isolates displayed extensive interactions between their aromatic bioremediation genes and peripheral genes, suggesting a strong integration of these genes into the broader metabolic network. For example, *Marinobacter* sp. CS14-16, with 10 aromatic genes, exhibited 5,926 interactions with other genes, underscoring its potential for involvement in complex metabolic processes. Similarly, *Ectothiorhodospira scottomollisae* sp. CS14-81 formed 2,838 interactions, demonstrating an intricate genetic network.

These findings suggest that bacterial isolates with robust gene interaction networks may be more efficient in bioremediation due to enhanced gene regulation and pathway integration. In contrast, isolates such as *Idiomarina* sp. OS2T-3 and *Idiomarina* sp. OS2T-6, which had only a single aromatic gene and lacked interactions among bioremediation genes, may have a more specialized or limited role in aromatic compound degradation. The lack of connectivity between bioremediation genes in some isolates, such as *Pseudoalteromonas* sp. CS1-48 and *Idiomarina* sp. OS2B-49, further indicates a weaker metabolic adaptation for degrading complex aromatic hydrocarbons. Overall, this study demonstrates that bacterial isolates with higher numbers of aromatic bioremediation genes, stronger interactions between those genes, and more extensive connectivity within the genetic network are likely to be more effective in bioremediation. These findings emphasize the importance of network complexity in predicting the metabolic potential of bacteria for environmental applications. Future studies should focus on validating these predictions through functional assays and exploring the regulatory mechanisms underlying these genetic interactions to optimize bioremediation strategies.

The limitations of this study include the 16S amplicon analysis of only six coastal sites sampled during a single period, which may limit the representation of spatial and seasonal variability of bacterial community across the Red Sea coastline, and the genomic analysis of only 21 previously recovered bacterial isolates. The predicted ARGs and aromatic-hydrocarbon-degradation potential of the isolates were not experimentally validated.

## 5 Conclusions

High bacterial diversity and richness were observed on the eastern coast of the Red Sea. Proteobacteria, Gammaproteobacteria, and Woeseiaceae predominated among the bacterial communities in the coastal sites. Specific biomarker taxa were identified across the sampling locations, highlighting differences between coastal regions. The detection of ARGs, particularly those associated with carbapenem resistance in the isolates, raises future public health concerns related to recreational beaches along the Red Sea. Aromatic compound bioremediation genes were identified in this study, suggesting that future research should incorporate metagenomics, transcriptomics, and metabolomics approaches to gain a deeper understanding of the functional roles of these microbial communities. Long-term monitoring is crucial for evaluating microbial responses to environmental changes, particularly in relation to climate change and human activities at various sites along the Red Sea. This would provide valuable insights into ecosystem stability, bioremediation potential, and microbial interactions in this distinct marine environment.

## Data Availability

The datasets presented in this study can be found in online repositories. The names of the repository/repositories and accession number(s) can be found below: https://www.ncbi.nlm.nih.gov/, PRJNA993259.
