# Supplementary material for: Diversity and functional genomic insights into antimicrobial resistance and aromatic hydrocarbon degradation genes in the Red Sea coast microbial community
Source: Front Microbiol. 2026 Sep 14;17:1936370. doi: 10.3389/fmicb.2026.1936370 (PMC13617036; doi:10.3389/fmicb.2026.1936370)
Supplement: Supplementary file 1 [file Supplementary_File_1.docx]

**Supplementary Data**

**Supplementary Figures and Tables**

**Figure S1.** Rarefaction analysis of sequence reads obtained from Red Sea coastal samples. Rarefaction curves of OTUs clustered at 97% sequence identity for collected samples.

**Figure S2.** Alpha diversity analysis of the Red Sea coast bacterial communities. (A) Chao1 estimate of richness, (B) observed species, (C) ACE index, and (D) Shannon index.

**Figure S3.** Multivariate analysis of the Red Sea coast bacterial communities. (A) Principal component analysis, and (B) hierarchical clustering using Bray-Curtis’ dissimilarity analysis showed variance among the sampling sites.

**Figure S4**. Beta diversity analysis in relation to (A) temperature, (B) pH, and (C) salinity using principal component analysis determined by the PERMANOVA statistical method.

**Figure S5.** Distribution of metal resistance protein (MRP) linked to the associated metal resistance cluster (MRC) detected in the genomes from the Red Sea isolates. The white boxes indicated that the respective genes were not detected.

**Figure S6.** Genetic networks of (A) *Marinobacter* sp. CS14-2, (B) *Cobetia* sp. CS14-12, (C) *Marinobacter* sp. CS14-16, and (D) *Idiomarina* sp. CS14-54. *Marinobacter* sp. CS14-16 has the highest aromatic gene count (10 genes, 5,926 interactions), while *Idiomarina* sp. CS14-54 has minimal connectivity (427 interactions).

**Figure S7.** Genetic networks of (A) *T. australica* sp. CS14-64, (B) *E. scottomollisae* sp. CS14-81, (C) *Cobetia* sp. CS19-23, and (D) *Halomonas* sp. CS19-36. *E. scottomollisae* sp. CS14-81 has the most complex network (788 nodes, 2,838 interactions), while Cobetia sp. CS19-23 and *Halomonas* sp. CS19-36 show moderate connectivity.

**Figure S8.** Genetic networks of (A) Vibrio sp. OS1T-47, (B) *Idiomarina* sp. OS2B-49, (C) *Halomonas* sp. OS2T-109, and (D) *Halomonas* sp. OS2T-176. *Vibrio* sp. OS1T-47 has strong interaction potential (1,097 interactions), while *Idiomarina* sp. OS2B-49 has the weakest network (402 interactions). *Halomonas* sp. OS2T-109 and OS2T-176 show greater complexity (>500 nodes).

**Figure S9.** Genetic networks of (A) *Roseovarius* sp. OS2T-32, (B) *Idiomarina* sp. OS2T-3, (C) *Idiomarina* sp. OS2T-6, and (D) *Halomonas* sp. OS2T-7. Roseovarius sp. OS2T-32 has the most connected network (600 nodes, 1,735 interactions), while *Idiomarina* isolates exhibit minimal connectivity (<500 interactions). *Halomonas* sp. OS2T-7 shows moderate complexity (514 nodes, 1,477 interactions).

**Table S1.** Genes related to aromatic bioremediation.

| **Gene** | **Enzymes/Products** |
| --- | --- |
| *benA* | Benzoate 1,2-Dioxygenase |
| *benB* | Benzoate 1,2-Dioxygenase |
| *benC* | Benzoate 1,2-Dioxygenase |
| *benD* | NAD-dependent dehydrogenase |
| *catA* | catechol 1,2-dioxygenase |
| *catB* | Muconate cycloisomerase |
| *catC* | Muconolactone D-isomerase |
| *pheA* | Muconolactone isomerase |
| *xylB* | D-xylulokinase |
| *xylH* | 4-Oxalocrotonate isomerase |
| *xylI* | 4-Oxalocrotonate decarboxylase |
| *xylJ* | 2-Oxopent-4-enoate hydratase |
| *xylG* | 2-Hydroxymuconic semialdehyde dehydrogenase |
| *xylF* | 2-Hydroxymuconic semialdehyde hydrolase |
| *dmpK* | phenol/toluene 2-monooxygenase (NADH) P0/A0  Phenol 2-monooxygenase, stimulatory component DmpM (UNIPORT) |
| *dmpM* |  |
| *dmpN* | **Phenol hydroxylase** |
| *dmpO* | phenol hydroxylase |
| *dmpP* | phenol hydroxylase |
| *nahG* | Salicylate hydroxylase |
| *nagK* | Fumarylpyruvate hydrolase |
| *pheA* | **Phenol hydroxylase** |

**Table S2.** Illustrates temperature, pH, and salinity at sampling sites of the Red Sea.

| **Sampling Sites** | **Temperature ℃** | **pH** | **Salinity %** |
| --- | --- | --- | --- |
| CS-4 | 27.1 ± 0.0 | 7.6 ± 0.01 | 3.1 ± 0.1 |
| CS-6 | 28.1 ± 0.0 | 7.9 ± 0.0 | 6.7 ± 0.0 |
| CS-7 | 28.4 ± 0.0 | 8.0 ± 0.0 | 10.0 ± 0.8 |
| CS-18 | 33.5 ± 0.0 | 8.0 ± 0.01 | 5.3 ± 0.2 |
| CS-20 | 33.0 ± 0.0 | 9.9 ± 0.03 | 5.1 ± 0.3 |
| CS-22 | 33.0 ± 0.0 | 8.0 ± 0.0 | 3.7 ± 0.0 |

**Table S3.** Genomic features of the sequenced bacteria from the Red Sea.

| **Isolates** | **Size (bp)** | **Contigs N50 (bp)** | **%GC** | **Contamination** | **Completeness** |
| --- | --- | --- | --- | --- | --- |
| *Pseudoalteromonas* sp. CS1-48 | 4290746 | 16068 | 40.8 | 5.5 | 99.9 |
| *Oceanisphaera* sp. CS12-67 | 3521088 | 138132 | 57.2 | 1.7 | 97.0 |
| *Ruegeria* sp. CS12-79 | 4550789 | 330927 | 57.9 | 3.3 | 97.2 |
| *Marinobacter* sp. CS14-2 | 4087744 | 23059 | 59.5 | 4.5 | 100 |
| *Cobetia* sp. CS14-12 | 4146766 | 140518 | 62.5 | 1.0 | 100 |
| *Marinobacter* sp. CS14-16 | 4362366 | 3511530 | 57.0 | 1.7 | 99.9 |
| *Idiomarina* sp. CS14-54 | 2,864,989 | 93,013 | 46.6 | 1.7 | 100 |
| *Oceanisphaera* sp. CS12-74 | 2024334 | 1136 | 57.0 | 11.5 | 95.8 |
| *T. australica* CS14-64 | 4681084 | 290470 | 53.9 | 14.4 | 99.7 |
| *E. scottomollisae* CS14-81 | 4369350 | 190207 | 61.1 | 0.7 | 98.7 |
| *Halomonas* sp. CS19-36 | 4795256 | 403225 | 54.9 | 1.3 | 99.7 |
| *Vibrio* sp. OS1T-47 | 5121346 | 1037237 | 44.8 | 0.0 | 100 |
| *Idiomarina* sp. OS2B-49 | 2445304 | 46290 | 47.1 | 1.3 | 99.6 |
| *Halomonas* sp. OS2T-109 | 4936927 | 87687 | 64.1 | 0.6 | 100 |
| *Cobetia* sp. CS19-23 | 2768891 | 1627 | 62.5 | 24.9 | 88.7 |
| *Halomonas* sp. OS2T-176 | 4887178 | 249055 | 63.8 | 1.0 | 100 |
| *Roseovarius* sp. OS2T-32 | 3861354 | 124389 | 63.0 | 2.1 | 100 |
| *Idiomarina* sp. CS19-60 | 2760277 | 19648 | 48.4 | 11.1 | 100 |
| *Idiomarina* sp. OS2T-3 | 2579375 | 294376 | 47.3 | 0.8 | 100 |
| *Idiomarina* sp. OS2T-6 | 2,598,750 | 515,170 | 47.3 | 0.0 | 100 |
| *Halomonas* sp. OS2T-7 | 4953610 | 154823 | 64.0 | 0.4 | 100 |

**Table S4.** The interaction of aromatic bioremediation genes with other genes in bacterial isolates.

| **Isolates** | **Aromatic Bioremediation Genes interaction with Aromatic Bioremediation Genes** | **Aromatic Bioremediation Genes interaction with Other Genes (hub or peripheral genes)** | **Other Genes (hub or peripheral genes) interaction with Other Genes (hub or peripheral genes)** | **Total nodes** | **Aromatic genes present in specific isolates** | **Hub genes** | **Peripheral genes** |
| --- | --- | --- | --- | --- | --- | --- | --- |
| *Pseudoalteromonas* sp. CS1-48 | 0 | 516 | 129482 | 518 | 2 | 256 | 260 |
| *Oceanisphaera* sp. CS12-67 | 28 | 3521 | 94997 | 460 | 8 | 188 | 264 |
| *Oceanisphaera* sp. CS12-74 | 0 | 209 | 21135 | 210 | 1 | 100 | 109 |
| *Ruegeria* sp. CS12-79 | 16 | 4114 | 213977 | 809 | 8 | 392 | 409 |
| *Marinobacter* sp. CS14-2 | 0 | 506 | 125155 | 507 | 1 | 245 | 261 |
| *Cobetia* sp. CS14-12 | 1 | 1045 | 110484 | 544 | 4 | 268 | 272 |
| *Marinobacter* sp. CS14-16 | 45 | 5926 | 166107 | 614 | 10 | 259 | 345 |
| *Idiomarina* sp. CS14-54 | 0 | 427 | 89314 | 428 | 1 | 208 | 219 |
| *T. australica* CS14-64 | 1 | 891 | 98694 | 450 | 2 | 202 | 246 |
| *E. scottomollisae* CS14-81 | 9 | 2838 | 202139 | 788 | 7 | 365 | 416 |
| *Cobetia* sp. CS19-23 | 1 | 598 | 39222 | 326 | 3 | 159 | 164 |
| *Halomonas* sp. CS19-36 | 10 | 2567 | 129976 | 539 | 5 | 262 | 272 |
| *Vibrio* sp. OS1T-47 | 1 | 1097 | 148610 | 557 | 2 | 270 | 285 |
| *Idiomarina* sp. OS2B-49 | 0 | 402 | 79508 | 403 | 1 | 188 | 214 |
| *Halomonas* sp. OS2T-109 | 3 | 1474 | 120276 | 513 | 3 | 249 | 261 |
| *Halomonas* sp. OS2T-176 | 3 | 1475 | 120506 | 514 | 3 | 252 | 259 |
| *Roseovarius* sp. OS2T-32 | 3 | 1735 | 162148 | 600 | 3 | 268 | 329 |
| *Idiomarina* sp. OS2T-3 | 0 | 424 | 88507 | 425 | 1 | 196 | 228 |
| *Idiomarina* sp. OS2T-6 | 0 | 425 | 88649 | 426 | 1 | 204 | 221 |
| *Halomonas* sp. OS2T-7 | 3 | 1477 | 120784 | 514 | 3 | 250 | 261 |
| *Idiomarina* sp. CS19-60* | - | - | - | - | 0 | - | - |

* No aromatic bioremediation genes were detected in *Idiomarina* sp. CS19-60
